# Risk prediction system for dengue transmission based on high resolution weather data

**DOI:** 10.1371/journal.pone.0208203

**Published:** 2018-12-06

**Authors:** Chathurika Hettiarachchige, Stefan von Cavallar, Timothy Lynar, Roslyn I. Hickson, Manoj Gambhir

**Affiliations:** 1 IBM Research Australia, Southgate, Victoria, Australia; 2 School of Mathematics and Statistics, The University of Melbourne, Parkville, Victoria, Australia; Beth Israel Deaconess Medical Center, UNITED STATES

## Abstract

**Background:**

Dengue is the fastest spreading vector-borne viral disease, resulting in an estimated 390 million infections annually. Precise prediction of many attributes related to dengue is still a challenge due to the complex dynamics of the disease. Important attributes to predict include: the risk of and risk factors for an infection; infection severity; and the timing and magnitude of outbreaks. In this work, we build a model for predicting the risk of dengue transmission using high-resolution weather data. The level of dengue transmission risk depends on the vector density, hence we predict risk via vector prediction.

**Methods and findings:**

We make use of surveillance data on *Aedes aegypti* larvae collected by the Taiwan Centers for Disease Control as part of the national routine entomological surveillance of dengue, and weather data simulated using the IBM’s Containerized Forecasting Workflow, a high spatial- and temporal-resolution forecasting system. We propose a two stage risk prediction system for assessing dengue transmission via *Aedes aegypti* mosquitoes. In stage one, we perform a logistic regression to determine whether larvae are present or absent at the locations of interest using weather attributes as the explanatory variables. The results are then aggregated to an administrative division, with presence in the division determined by a threshold percentage of larvae positive locations resulting from a bootstrap approach. In stage two, larvae counts are estimated for the predicted larvae positive divisions from stage one, using a zero-inflated negative binomial model. This model identifies the larvae positive locations with 71% accuracy and predicts the larvae numbers producing a coverage probability of 98% over 95% nominal prediction intervals. This two-stage model improves the overall accuracy of identifying larvae positive locations by 29%, and the mean squared error of predicted larvae numbers by 9.6%, against a single-stage approach which uses a zero-inflated binomial regression approach.

**Conclusions:**

We demonstrate a risk prediction system using high resolution weather data can provide valuable insight to the distribution of risk over a geographical region. The work also shows that a two-stage approach is beneficial in predicting risk in non-homogeneous regions, where the risk is localised.

## Introduction

Dengue is a viral infection that is endemic in over 100 countries, primarily in tropical and sub-tropical regions [1]. Dengue viruses are primarily maintained in a human-to-mosquito-to-human cycle, hence mosquitoes are the “vector” of the disease. These viruses are transmitted by mosquitoes of the genus *Aedes*, primarily by *Aedes aegypti* and secondarily by *Aedes albopictus*. Dengue is the fastest spreading vector-borne viral disease, resulting in 40% of the world’s population living in an area at risk [2]. Dengue infections are massively under-reported and also masked by symptomatically similar illnesses [3]. There has been a 30-fold increase in the number of dengue cases over the last 50 years [4]. The World Health Organisation (WHO) currently estimates there may be 50–100 million dengue infections worldwide every year. However, Bhatt *et al*. [5] estimates this to be 390 million dengue infections (95% confidence interval 284-528 million), of which 96 million (95% confidence interval 67-136 million) manifest clinically (with any severity of disease). The case-fatality rate is usually lower than 1%, but in the absence of prompt diagnosis and proper treatment it can be as high as 20% [6]. There is no specific antiviral to treat dengue, and although a vaccine has been registered [7], its use has generated controversy (see, for example, [8]). The primary preventive measure to reduce dengue infections is the control of mosquito populations.

A risk prediction system for an infectious disease can help in many ways, including prevention and preparedness. Dengue is primarily transmitted by *Aedes aegypti* mosquitoes and hence breaking the human-to-mosquito-to-human cycle by controlling the *Aedes aegypti* population reduces dengue incidence. The relationship between dengue incidence and weather attributes is well-established, as described later in section, hence a dengue risk-prediction system based on the relationship between the *Aedes aegypti* mosquito population and weather attributes appears prudent. Such a risk prediction system would be of substantial benefit in controlling dengue via reducing/eliminating the transmitting mosquitoes. There are limited analyses establishing the relationship between the *Aedes aegypti* mosquitoes and weather attributes, as further outlined in the last paragraph of the Introduction. The existing risk prediction models for dengue are based on the relationship between the weather attributes and the dengue incidence, but not the mosquitoes [9–12]. Furthermore, these models do not incorporate high-resolution weather data.

We demonstrate that we can use easily accessible high resolution weather data to construct a risk prediction system for dengue. This system allows the user to identify geographical regions where the *Aedes aegypti* mosquitoes are present or absent, and hence where transmission risk of dengue exists. Further analysis is conducted on geographical areas with a high probability of presence of *Aedes aegypti* mosquitoes to estimate the population numbers. This can be interpreted as an estimate of the magnitude of dengue transmission risk, and informs further modelling efforts to establish the efficacy of control strategies. We illustrate our proposed approach using mosquito related and weather related data collected in Taiwan. Note that to fully understand the risk of dengue transmission both the mosquito and human population features must be taken into account. However in this paper we use “dengue risk” to refer to the mosquito attributable risk of dengue transmission posed only by *Aedes aegypti*, and specifically as represented by their larvae.

The relationship between dengue incidence and weather attributes is well-established by many studies that have assessed this complex relationship [13–23]. These studies do not use high temporal resolution weather data and instead use weekly [23–26], monthly [13, 16–18, 21], or annual data [27, 28]. Weather has been identified as an effective predictor for dengue fever by a time series analysis on the occurrence of dengue cases in Kaohsiung, Taiwan [17]. This work shows that, based on cross-correlations, the incidence has most significant associations with maximum monthly temperature, minimum monthly temperature, relative humidity, and monthly rainfall, at a lag of 2 months. Campbell *et al*. [13] determined that temperature and humidity is correlated to the incidence, but not the amount of rainfall while Vu *et al*. [29] found that temperature, humidity, sunshine and rainfall has significant associations with dengue incidence.

Vector surveillance is a routine practice in many dengue-endemic countries and is recommended by the WHO [30]. This is used to determine changes in geographical distribution of vectors, for monitoring and evaluating control programmes, for obtaining relative measurements of the vector population over time, and for facilitating appropriate and timely decisions regarding interventions. Many studies have been conducted on finding the relationship between entomological indices and dengue incidence [19, 31–38]. Furthermore, the biological causation is well established between entomological indices and dengue cases despite some studies suggesting no statistically significant relationship [37, 38], possibly due to practical hindrances. One possibility for not identifying such a relationship may be that it can be masked by the use of large geographical areas resulting in key dengue hotspots with high vector indices being demeaned by neighbouring areas with a low vector density.

The relationship between mosquito populations and weather dynamics have been studied, but less extensively than the relationship between dengue incidence and weather. Yang *et al*. [39] have shown that the presence of *Aedes aegypti* is a prerequisite to initiate and establish an outbreak. Yang *et al*. [40, 41] have shown the effect of temperature in lab conditions on key aspects of the *Aedes aegypti* adult and aquatic lifecycle. A longitudinal study of *Aedes aegypti* in San Juan city, Puerto Rico by Barrera *et al*. [19] indicated significant effects of rainfall and temperature on the average number of females per trap per day. Tsai *et al*. [42] conclude that there may be a sharp inflation in the mosquito population, seven days after a period of intense rain, if the weather remains warm and humid. They also mention that this may not be an immediate impact of the rainfall, but its contribution to maintain humidity is preferred for larvae to survive.

Tsai *et al*. [43] showed *Aedes aegypti*, but not *Aedes albopictus*, and human population density in southern Taiwan are closely associated with an increased risk of local dengue incidence. Their study used samples of mosquito larvae from 7,019 subtownships (that is, the smallest administrative unit in this study) on the main island of Taiwan between 2009 and 2011.

## Materials and methods

### Materials

We conduct a statistical analysis of mosquito and weather data to determine the risk of dengue transmission in the main island of Taiwan due to *Aedes aegypti* mosquitoes.

We consider data collected in the main island of Taiwan in this study (22-26°N and 118-122°E). The total area of the island is 36,193 km^2^. Taiwan is oriented in a south-to-north direction across the Tropic of Cancer, such that its north part belongs to sub-tropical climate zone, while the south part belongs to the tropical climate zone. The country consists of 22 second level administrative divisions (referred to as “divisions” henceforth). In this study we consider only the main island of Taiwan which consists of 19 divisions. Dengue is not endemic in Taiwan and the importation of the virus from neighbouring countries initiates local outbreaks [44, 45]. Furthermore, dengue incidence in Taiwan is not distributed evenly across the country, with a majority of cases being concentrated to some geographical regions [46].

We use mosquito data collected as a part of national routine entomological surveillance of dengue by the Taiwan Centers for Disease Control (CDC) across Taiwan. These mosquito related data are collected by local health departments in the community, then aggregated and integrated into a single database by Taiwan CDC. The data is collected by local health departments in an impromptu manner where the officials visit inside and outside the dwellings and count the number of water containers and the number of larvae in them, if there are any, and visible adult mosquitoes etc. Implementation of most vector surveillance efforts becomes more intensive once dengue cases are reported or confirmed. Hence, the mosquito numbers when and where there are no dengue cases may be underreported. For the same reason the mosquito numbers in less urbanized areas may also be underreported. We primarily consider the data collected on *Aedes aegypti* larvae within a year from January, 2012. We have only considered *Aedes aegypti* here as they have very different ecological footprint compared to *Aedes albopictus*, hence they require different models. Furthermore, it has been shown that *Aedes aegypti* are the most competent vector for transmitting dengue, particularly in Taiwan [42]. For our study, we use the observed numbers of *Aedes aegypti* larvae and the number of observed containers (both inside and outside). The observed number of adult mosquitoes were quite sparse, therefore we proceeded with the observed larvae, which is an earlier stage of the mosquito life cycle. The observed numbers of *Aedes aegypti* larvae are inherently noisy with a large number of zeroes and positively skewed distribution with a high variance. A summary of the distribution of larvae is shown in the Supplementary materials (S1 Fig). For each data collection occasion the date of collection and the geospatial location of the region in which the data were collected are available. We use these data to integrate the entomological data with the weather attributes.

We perform simulations using IBM’s Containerized Forecasting Workflow, a high spatial- and temporal-resolution forecasting system, which is based, in part, on the Advanced Research WRF (ARW) core of the Weather Research and Forecasting (WRF) model. This produces high resolution weather data, with grid spacings of 10 km and hourly output for the specified period, in this case the year 2012. Regarding the physics schemes used, the Containerized Forecasting Workflow was executed using the the Yonsei University (YSU) [47] planetary boundary layer (PBL) scheme, the WRF Double-Moment 6-Class Microphysics Scheme (WDM6) [48] and with the Rapid Radiative Transfer Model (RRTM) [49] long-wave radiation sand New Goddard short-wave scheme [50].

We used NOAA High-resolution Blended Analysis of Daily SST and Ice (OI SST V2) and NCEP FNL (Final) Operational Global Analysis data was used for model initialisation and boundary conditions [51]. Observations from NCEP ADP Global Upper Air Observational Weather Data and Surface Observational Weather Data were utilised for forcing the simulation towards observations. The simulation is forced towards spatiotemporally relevant observations. This improves the accuracy of the simulation but produces edge effects.

There were some gaps in the simulation. We only consider the days where data for all 24 hours are available to avoid bias, hence discarding data for 31 days, including 19 days in December and 5 days in January. Based on hourly weather data we compute the corresponding daily values, for example the maximum of the temperature values across 24 hours is considered the daily maximum temperature. We consider the following clusters of related weather variables and then we select a single variable from each cluster for our analysis to avoid collinearity issues. A variable from each cluster is selected based on the Akaike Information Criterion (AIC) and the accuracy of the models.

- Minimum/maximum/average temperature—derived from the hourly temperatures measured at 2m above the ground across 24 hours (in *K*).
- Minimum/maximum/total precipitation—derived from the hourly sum of accumulated grid scale precipitation and accumulated cumulus precipitation over 24 hours (in *mm*).
- Minimum/maximum/average relative humidity—derived from the relative humidity at each hour of the day.

In addition to the three variables selected from above clusters we use the terrain height at the grid point (in *meters*) for our model.

### Methods

**Fig 1:**
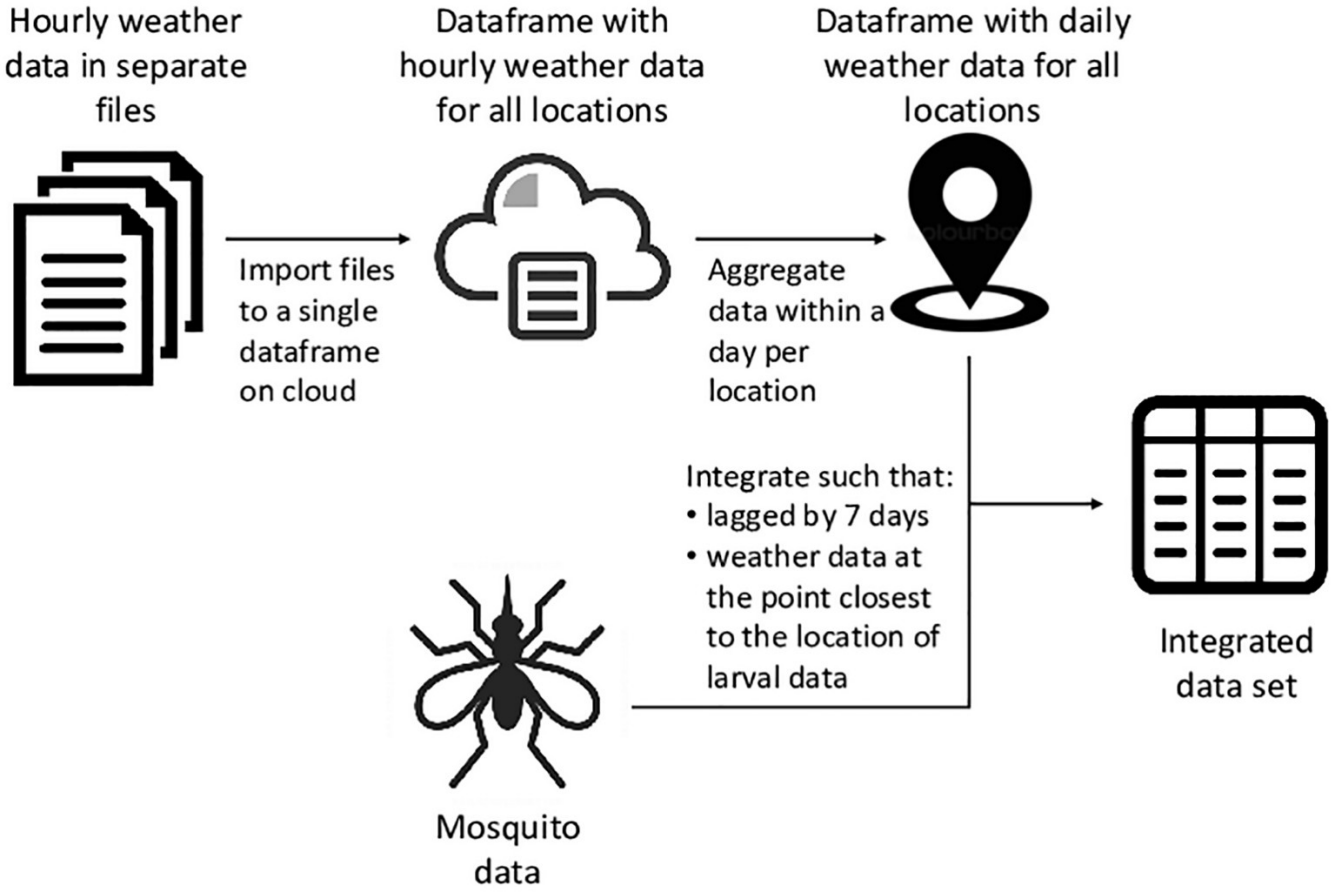
A schematic representation of the data pipeline. This depicts aggregation of the weather reanalysis data and integration with the mosquito data.

We integrate the mosquito and weather data using the temporal and spatial stamps. This is done by identifying the spatially closest reanalysis weather data point available inside the main island of Taiwan to the larval data via euclidean distance, with a time lag of seven days. The distances between larval and weather data location varied between 0.03km and 7.32km with a mean of 3.55km. The time lag of seven days is used due to estimates of population increases peaking then [42]. A schematic of this data aggregation and integration process is shown in Fig 1. The integrated dataset consists of 39,752 entries.

We chronologically split the dataset into training and test sets. We use all the data collected before 23-09-2012 (75% approx.) for fitting the model (*training set*) and the remaining data for the validation (*test set*) for each stage of the statistical analysis. While the k-fold cross validation is one of the most widely used methods for model evaluation, we do not incorporate it since we use time-series data in our work. Due to inherent serial correlation and potential non-stationarity of the data the application of k-fold cross validation is not straightforward. In the forecasting literature, out of sample evaluation is the standard evaluation procedure [52]. Furthermore, we considered our dataset is sufficiently large (39,752 observations) to perform an out of sample evaluation.

**Fig 2:**
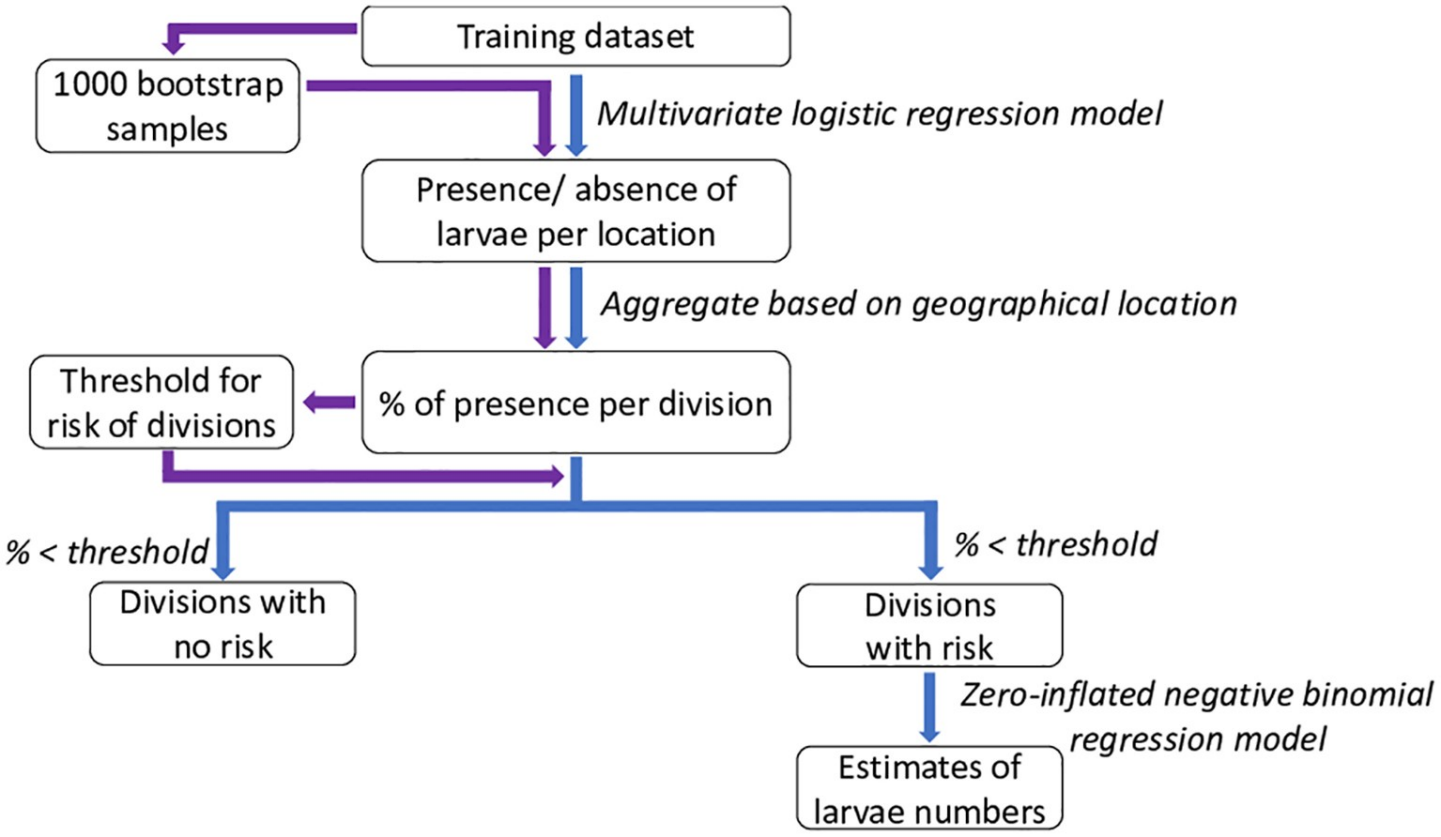
A schematic representation of the statistical approach. The path in blue represents the main two-stage approach, where in stage 1 presence or absence of *Aedes aegypti* larvae is predicted, and in stage 2 the number of larvae are estimated for the divisions classified as larvae-positive. The path in purple represents the bootstrap approach followed to determine the threshold value for classifying the divisions.

The level of dengue risk depends on the vector density, hence estimating the vector density is our ultimate aim. Recall that, by “dengue risk” we refer to the mosquito attributable risk of dengue transmission posed only by *Aedes aegypti*. To improve the accuracy of the density prediction, the statistical analysis is conducted in two stages, as outlined in Fig 2. In stage 1 of the statistical analysis, we use information on all available locations and predict whether *Aedes aegypti* larvae are present, based on weather inputs. The predicted larval status of locations are aggregated at the second level administrative divisions, to identify the probability of *Aedes aegypti* presence in each division. This probability can be considered as an indication of the level of risk in each division. We then use a bootstrap approach to determine the threshold level to determine whether a division is at risk of dengue transmission, and hence considered for further analysis in stage 2. In stage 2 of the statistical analysis, we estimate the number of larvae in the larvae positive counties identified in stage 1 based on weather inputs which provides an indication of the potential for transmission. We explain the analyses in stage 1 and stage 2 in the following two sections respectively.

#### Stage 1: Predicting the mosquito presence

We performed a multivariate logistic regression to fit a model for the presence/absence of larvae. The response variable was defined such that it equals 1 if the number of larvae is non-zero and equals 0 otherwise. Biologically, the number of larvae reported depends on the number of containers found for a given sample. Therefore, we use the number of containers collected in each sample as an offset variable to reduce this effect.

We first fit logistic regression models with each of the single variables with the number of containers as an offset variable to select a variable from each cluster of variables described in the materials section. Variables were selected based on the AIC and the percentage of correct predictions over all predictions (the accuracy). We then considered these selected variables, maximum temperature, maximum precipitation, average relative humidity, terrain height and all possible two-way interaction terms as candidate predictor variables for the logistic regression model, using the number of containers as an offset variable. A two-way stepwise selection method was used to determine which individual variables and two-way interaction terms should be included in the model based on AIC. This logistic regression model outputs the probability that the larvae is present at the location of the sample being collected on the day it is been collected. The probability level which maximises the sum of specificity and sensitivity is used as the threshold level to classify the output as larvae positive or larvae negative.

We labelled each location in our integrated dataset as larvae positive or larvae negative based on the fitted logistic model. Then the percentage of larvae positive locations within each division is computed. This percentage itself can serve as an indicator of mosquito attributable risk of transmission for a division. However, taking a further step forward, we determined a threshold level which allows us to label a division as at risk or not. The data collected for these divisions at risk are considered for stage 2.

We used the same training set for determining the threshold level to classify a division at risk. We drew 1000 bootstrap samples of the same size as the training set with replacement. These samples were drawn such that the proportion of observations per division in the bootstrap sample is similar to that of the training set. Then the process in stage 1, that is fitting a logistic regression model and determining the percentage of larvae positive locations for the divisions, is repeated for the 1000 samples. We label divisions with at least 1% of observed larvae positive locations as at risk. We assumed that if the percentage of observed larvae positive locations is below 1%, they are likely due to noise or error, such as data collection or data entry errors. We determined the optimum threshold value for classifying a division as at risk using these labels as the target variable and the percentage of predicted larvae positive locations for the divisions for 1000 bootstrap samples as the predicted variable. Specifically, we considered the percentage which maximises the sum of sensitivity and specificity as the threshold value.

#### Stage 2: Estimating the number of Aedes aegypti mosquitoes

The number of larvae in the divisions with risk determined in stage 1 are positively skewed, overdispersed, and due to the nature of the data collection process, have a large amount of zeroes. This suggests a zero-inflated negative binomial regression is suitable to model the relationship between the number of larvae and the weather-related predictor variables. We also modelled the relationship using negative binomial, Poisson and zero-inflated Poisson regression models, to determine the best fitting model. The models were compared using the Vuong’s closeness test, which is a likelihood-ratio-based test for model selection using the Kullback-Leibler information criterion. The zero-inflated negative binomial model outperforms the other models and hence our reported estimates of larvae numbers are based on this model. A comparison of the models is shown in the Supplementary Materials (S1 Table).

We used the same set of predictor variables used in the logistic regression model discussed in stage 1 as candidate predictor variables here. A two-way stepwise selection method was used to determine which variables should be included in the model based on AIC. The zero inflated negative binomial regression model assumes that there are two distinct data generation processes which generates structural zeros and a process which generates counts, some of which may be zero. Hence it is a combination of two models, one is a binary model to model which of the two processes the zero outcome is associated with, the other is a negative binomial model to model the count process.

We calculated 95% prediction intervals for each observation in the training set using a bootstrap method. In this process 1000 sets of regression coefficients for a zero-inflated negative binomial model with the same variables were simulated such that they follow a multivariate normal distribution with the mean and variance-covariance matrix being equal to the regression coefficients of the fitted model and their variance-covariance matrix. Then we predicted the number of larvae using each set of regression coefficients per every observation. This process results in 1000 predicted values for each observation. Then prediction interval for an observation is defined as the 2.5^th^ and 97.5^th^ percentiles of the 1000 predicted values.

## Results

### Stage 1: Mosquito presence

For the stage 1 analysis to predict *Aedes aegypti* larvae presence in a location, all of the predictor variables and their two-way interaction terms were significant except the interaction between average relative humidity and terrain height. In Table 1, we present the partially standardised regression coefficients of the predictor terms in the model to compare their relevance. We use the simple and straightforward Agresti approach to find the standardised coefficients where the coefficient is specified in ‘per standard deviation’ unit of the predictor [53, 54]. The average relative humidity, maximum temperature and the terrain height has a large influence on the probability of larvae existence, individually and collectively.

**Table 1:** Standardised coefficients of the terms in the logistic regression model to predict presence of *Aedes aegypti* larvae.

Term	Standardised Coefficient	95% CI
Average relative humidity	-12.34	(-12.78, -11.91)
Maximum temperature	-10.35	(-10.47, -10.24)
Terrain height	8.70	(8.68, 8.73)
Maximum precipitation	1.67	(0.75, 2.60)
Maximum temperature×Average relative humidity	12.50	(12.50, 12.50)
Maximum temperature×Terrain height	-9.30	(-9.30, -9.30)
Maximum precipitation×Terrain height	-4.19	(-4.19, -4.19)
Maximum temperature×Average relative humidity	-2.14	(-2.14, -2.14)
Maximum temperature×Maximum precipitation	-1.58	(-1.59, -1.58)

**Fig 3:**
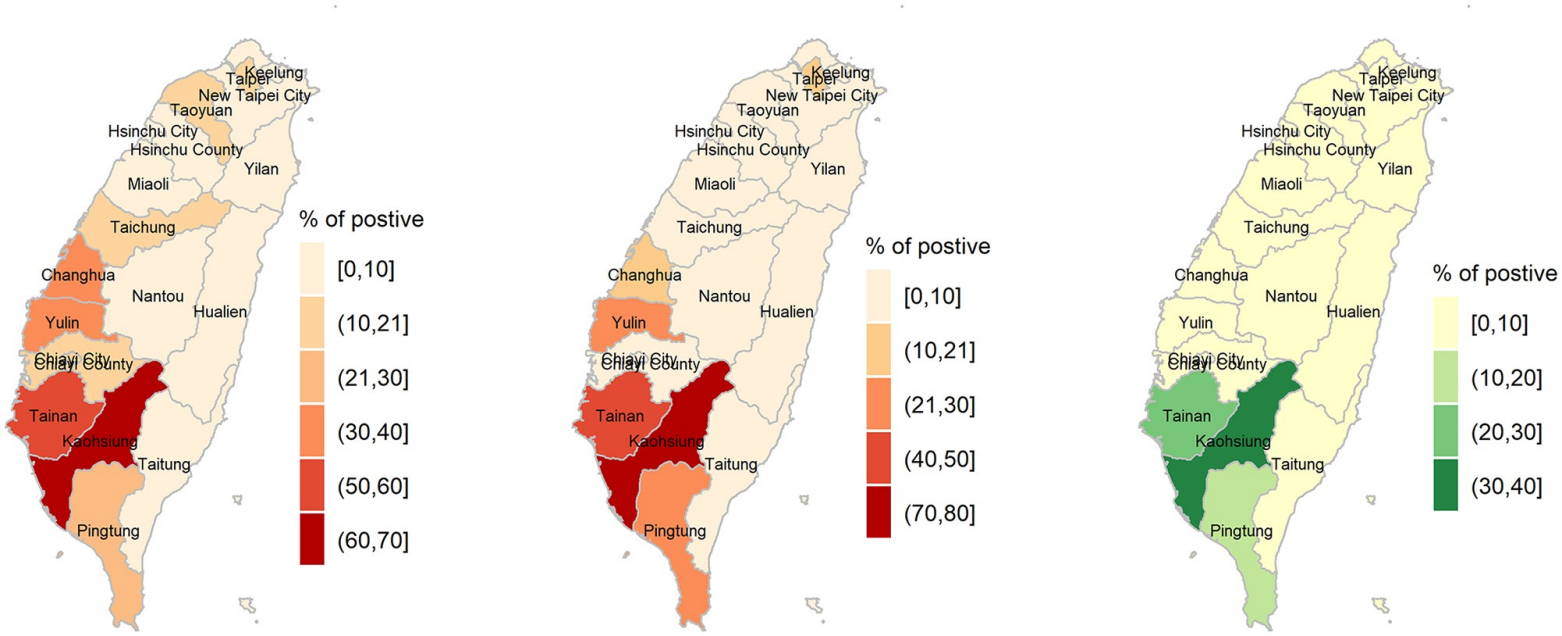
Maps of the percentages of *Aedes aegypti* larvae presence resulting from stage one of the risk prediction. Subfigures (a) shows predicted percentages for training set, (b) shows the predicted percentages for test set and (c) shows the observed percentages of positive locations within a division. The optimal threshold to classify a division as at risk is 21% for the training and test sets, hence the first two colour bands are for the divisions with no risk.

The threshold level of 0.179 maximised the sum of sensitivity and specificity of the diagnosis with sensitivity of 0.83 and specificity of 0.68, and an overall accuracy of 0.71. The percentage of larvae positive locations in the divisions is presented in Table 2. The test set delivered a sensitivity of 0.71 and specificity of 0.71, and an overall accuracy of 0.71. The area under the curve (AUC) for the training and test sets were 0.76 and 0.71 respectively. The Wilcoxon test revealed that the order of the percentages of larvae positive locations in divisions in the training and test sets is not statistically different. In Fig 3(a) we show the predicted percentages of larvae positive locations in the divisions in the training set, followed by the test set in Fig 3(b). The observed percentages of larvae positive locations in the divisions is shown in Fig 3(c). There exists a discrepancy between the observed and estimated percentages of larvae positive locations in the divisions. This is partly due to the lower specificity of the fitted model and due to the nature of mosquito existence and inefficient data collection process. While the weather attributes do not vary much in the close proximity, the data may still show differences in the larvae numbers. This is likely due to biases in data collection in heavily (human) populated areas, as well as the result of heavily populated areas having more of the artificial breeding sites. Note that other factors such as urbanisation [46] and availability of artificial water containers [55] has significant influence on the *Aedes aegypti* population and the existence of similar weather attributes does not imply similar probability for mosquito prevalence.

**Table 2:** Percentage of larvae positive locations in each division.

Division	% of larvae postive locations	Number of observed containers
Kaohsiung City	66.5%	699070
Tainan City	54.1%	121266
Yunlin County	39.5%	10770
Changhua County	32.6%	12747
Pingtung County	24.6%	105486
Chiayi City	21.8%	14258
Taichung City	17.6%	23706
Chiayi County	16.9%	11074
Taipei City	15.5%	49385
Taoyuan City	12.3%	27158
New Taipei City	2.7%	21530
Hualien County	2.2%	6456
Miaoli County	2.1%	7619
Hsinchu City	1.9%	8864
Keelung City	0.9%	1700
Hsinchu County	0.4%	5398
Nantou County	0.2%	10335
Taitung County	0%	18560
Yilan County	0%	8180

### Threshold level to classify an administrative division at risk

**Fig 4:**
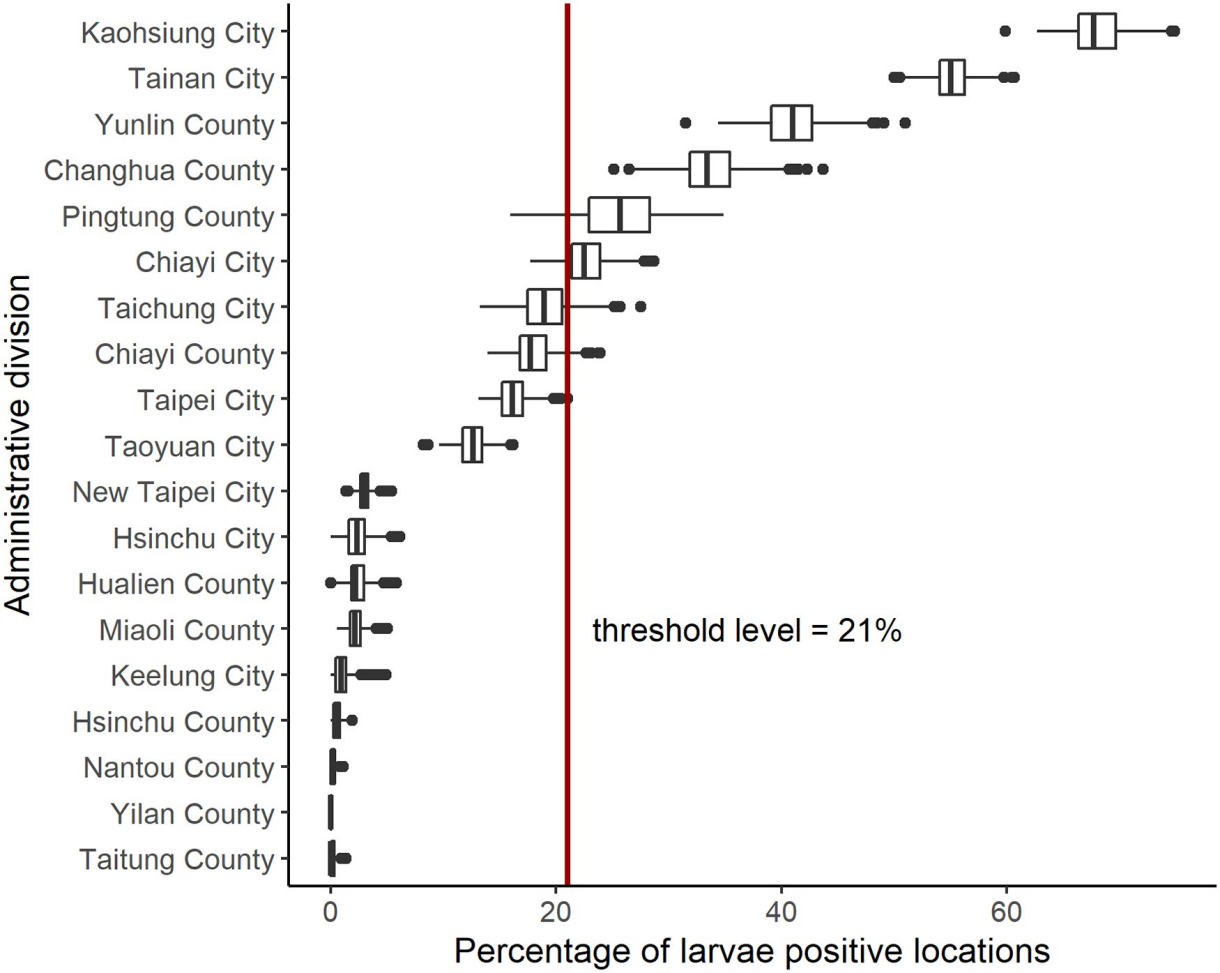
Boxplots for the distribution of the percentage of larvae positive locations resulted in the 1000 bootstrap samples by division. The threshold level determined based on these results is 21%, and shown by the vertical line.

The boxplots for the percentages of larvae positive locations per division resulted from 1000 bootstrap samples are shown in Fig 4. Based on these results 21% was determined as the optimum value to classify a division as at risk, with a sensitivity of 0.96, a specificity of 0.81, and an overall accuracy of 0.84. The divisions Kaohsiung city, Tainan city, Yunlin county, Changhua county, Pingtung county and Chiayi city had percentages of larvae positive locations above 21% (Fig 3(a)). Therefore the number of larvae in these divisions was estimated in our stage 2.

### Stage 2: Mosquito numbers

Stage 2 of the risk prediction estimates the number of larvae in each division, using the two-way stepwise selection procedure. The statistically significant predictors were found to be maximum temperature, maximum precipitation, average relative humidity, and terrain height for both the count and predicting excess zeroes. The overdispersion of the data and suitability of a negative binomial model for the counts was confirmed by finding a statistically significant value of the dispersion parameter (*θ*) of 0.085. We present the partially standardised regression coefficients of the predictor terms of the count model and the zero-inflation model in Table 3 to compare their relevance.

**Table 3:** Standardised coefficients of the terms in the logistic regression model to estimate number of *Aedes aegypti* larvae.

Term	Standardised Coefficient	95% CI
Terms in the count model (negative binomial with log link)		
Log(theta)	-136.62	(-136.69, -136.62)
Maximum temperature	14.68	(14.66, 14.69)
Average relative humidity	13.01	(13.00, 13.02)
Terrain height	-11.93	(-11.93, -11.93)
Maximum precipitation	1.83	(1.82, 1.83)
Terms in the zero-inflation model (binomial with logit link)		
Maximum temperature	-15.85	(-15.88, -15.81)
Average relative humidity	-6.86	(-6.88, -6.83)
Terrain height	-4.73	(-4.74, -4.72)
Maximum precipitation	-4.00	(-962.44, 954.44)

**Fig 5:**
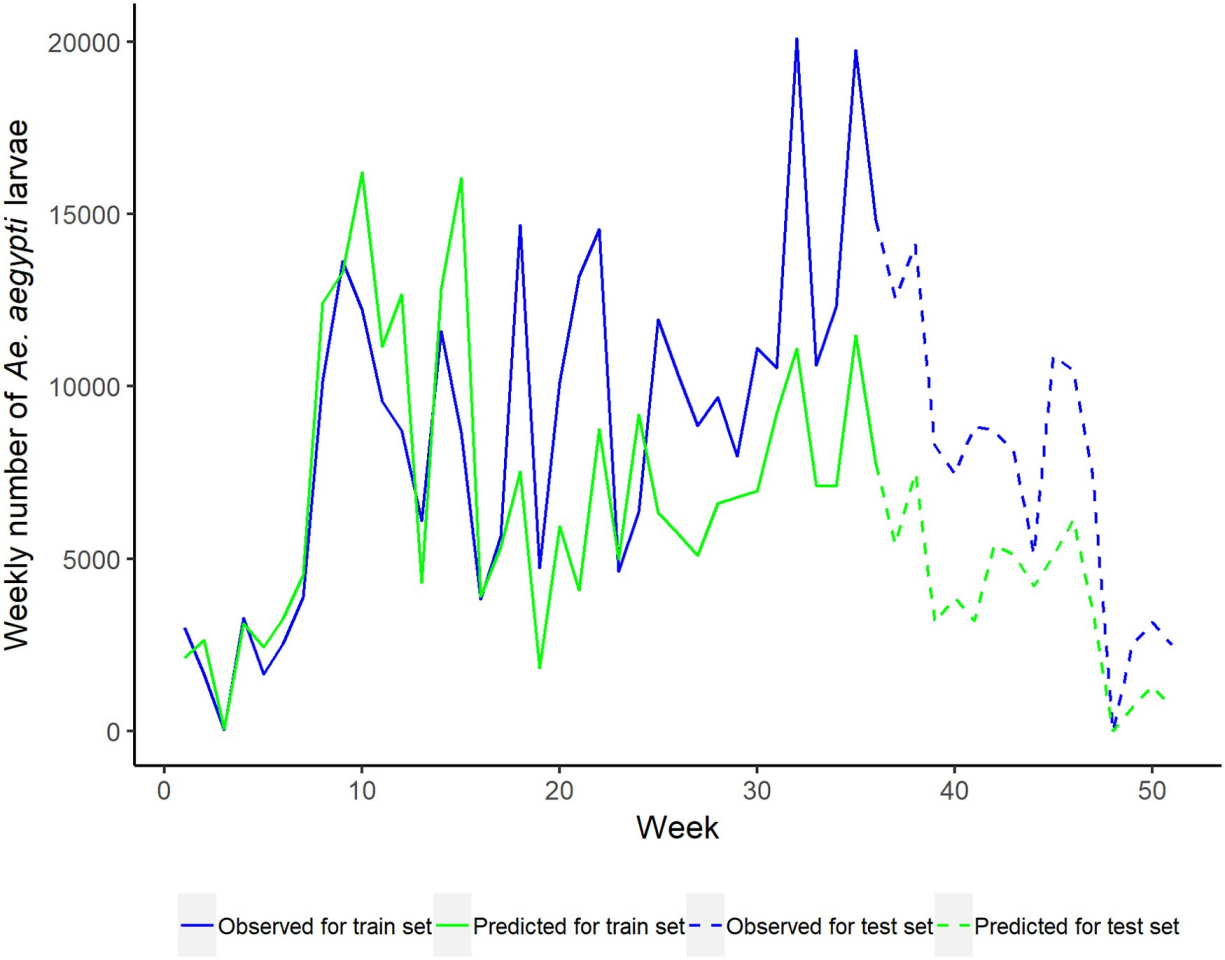
Total number of *Aedes aegypti* larvae in the at risk divisions by week. The blue colour is used to denote observed value and the green colour for the predicted values. The solid line is for the training set and the dashed line is for the test set.

The nominal 95% prediction intervals of larvae numbers produced satisfactory coverage, containing 98.18% of the observations for the training set and 96.36% for the test set. We have shown the prediction intervals for the observations in the Supplementary Materials (S2 Fig). The mean squared error was 1809.9 for the training set compared to the 2364.6 for the test set. To visually inspect the observed and predicted values, we plot the weekly sum of *Aedes aegypti* larvae in the divisions we classified as at risk in Stage 1, in chronological order in Fig 5. The blue line denotes the observed larvae numbers and the green line denotes the predicted values using the fitted regression model. The solid and dashed lines represent the training and test sets respectively. This suggests the model fits the data well for most weeks, and often follows the pattern of the actual sum of larvae counts. We do not show the prediction intervals here since the values we show here are the sum of predicted values. Instead, we show the prediction intervals for observations in the Supplementary Materials (S2 Fig) as mentioned above.

## Discussion

We demonstrated a risk prediction system for dengue risk in endemic countries that uses easily-generated, high resolution and big weather data. This framework should permit public health authorities to determine the administrative/geographical regions on which to focus intervention strategies and vector surveillance.

We have identified predictors and their relationship to presence and subsequently the number of *Aedes aegypti* larvae in a statistically robust way. The stage one risk prediction identified the following predictors as significant in determining whether *Aedes aegypti* larvae are present: maximum temperature, maximum precipitation, average relative humidity, precipitation status, terrain height and their two-way interactions, except the interaction between the the average relative humidity and terrain height. According to the results of stage one, Kaohsiung city and the Tainan city had the highest proportion of larvae positive locations. The stage two risk prediction confirmed the suitability of a zero-inflated negative binomial regression model to estimate the larvae counts for locations where the stage one analysis predicted their presence. Furthermore, the maximum temperature, precipitation status, average relative humidity and terrain height were identified as significant variables affecting the larvae counts in identified larvae positive regions. Even though other machine learning techniques such as support vector machines and random forest models can be incorporated into a similar two-stage approach, and may even enable improved predictions, we would lose the interpretability of the model making it difficult to gauge the relationship between the individual predictor variables and the response variable.

The regression models fitted in stage 1 and 2 both suggest that the average relative humidity and the maximum temperature has the highest impact on both the existence of larvae and their counts. This corresponds with the findings of Wu *et al*. [17], Campbell *et al*. [13], and Vu *et al*. [29] that the temperature and humidity has most significant association with the dengue incidence. Furthermore, Yang *et al*. [39], Barrera *et al*. [19], and Tsai *et al*. [42] have revealed the significant effects of temperature and humidity on the mosquito population. Wu *et al*. [17] and Campbell *et al*. [13] identified rainfall as having a strong correlation with the dengue incidence while Barrera *et al*. [19] identified also indicated the significant effect on the mosquito population. Our models also identify that rainfall has a significant effect on both the existence of larvae and their counts, even though less strong than the impact of temperature and humidity.

Our risk prediction system utilises a two-stage approach. The mosquito data we use here is noisy, and using a two-stage approach helps us minimise the effect of this noisiness. In our approach the logistic regression in stage one classifies the larvae positive locations with an overall accuracy of 0.71 (sensitivity of 0.83 and specificity of 0.68), whereas a zero-inflated negative binomial regression model to the full data set directly identifies the larvae positive locations with an overall accuracy of 0.42 (sensitivity of 0.99 and specificity of 0.28). Here, we should note that the zero-inflated negative binomial regression model is not usually used for classification, but for estimating expected counts. Further, the mean squared error of a zero-inflated negative binomial regression model which is directly applied to the dataset results in an 9.6% increase over the mean squared error produced by this two-stage approach. Campbell *et al*. [22] use a similar two-level approach in their work. They use weather data by district and week (2005-2012) as inputs and predict districts in which dengue virus transmission occurred, and the intensity of transmission on a scale of 1 to 5, using a binary classification tree technique.

The mosquito data we use here may not reflect the true relationship between the existence of mosquitoes and the weather attributes. Data collection for mosquitoes is known to be difficult and error prone. The mosquito numbers may be biased due to several reasons, including difficulties with opportunistic sampling bias. Implementation of most vector surveillance efforts become more intensive once dengue cases are reported or confirmed. Hence, the mosquito numbers when there are no dengue cases may be underreported. Furthermore, the mosquito reduction intervention varies over time, which would cause a change in mosquito numbers that is not weather-induced. Our results are therefore limited by these issues. However, this modelling approach has identified likely explanatory variables of mosquito populations, which could aid future mosquito surveillance design, which could in turn refine the modelling. Also, the weather data were not available below 22.3°N, therefore, the data for the bottom part of Pingtung county was not considered for the analysis. This may have an impact on the overall percentage of larvae positive locations of Pingtung county. Furthermore, due to unavailability of the full weather data, data for some days had to be discarded, out of which the majority of the days were in December. This results in a lower number of larvae, both observed and predicted, than actually present. There are two and perhaps three apparent clusters of divisions that can be seen in Fig 4. The absence of *Aedes aegypti* larvae is close to certain in the final nine divisions (New Taipei city, Hsinchu city, Hualien county, Miaoli county, Keelung city, Hsinchu county, Nantou county, Yilan county, and Taitung county). The presence of *Aedes aegypti* larvae in the first two divisions (Kaohsiung city and Tainan city) is also certain. However, the classification of the middle divisions is less clear, particularly around the threshold line, and hence future intensive mosquito surveillance studies may need to focus more on this group.

On another note, we split our dataset for training and test sets in a chronological order. However, we do not fit a dynamic time-series model in our approach and hence do not capture seasonal variations. Moreover, we only have a years’ worth of data where the training set consists of data only for the first 9 months of the year. Consequently we do not use the mosquito data in the months with the highest dengue incidence. This results in underestimated mosquito numbers for our test set. Also, in this work we have not considered the time variation in mosquito numbers. Our risk prediction approach can be repeated for smaller time periods, such as a month or a quarter, if the larval data are sufficient. A time-sensitive analysis would enhance the strength of the approach to help time mosquito intervention programs more effectively.

In principle, the ecological footprint of mosquitoes should be similar in different countries. Therefore externalising the relationships and findings established by this work to other countries in more or less sophisticated methods is plausible. Rogers *et al*. [56] and Hay *et al*. [57] have successfully applied the relationship between mosquito attributes and climate established for one geographical region to other regions. However, this requires further study to determine proper techniques for extending the relationships and evaluate its suitability.

The results of this analysis can inform where further investment in mosquito control interventions on transmission and mosquito surveillance will have the most impact to understanding and predicting the dengue dynamics. Furthermore, we have outlined a framework for predicting risk of dengue transmission in any country where mosquito surveillance occurs and high-resolution weather data are available.

## Supporting information

S1 Fig Cumulative density plot of the observed numbers of *Aedes aegypti* larvae on the main island of Taiwan. The observed numbers of *Aedes aegypti* larvae consist of a large number of zero observations and it follows a positively skewed distribution with a high variance. The minimum and median of the counts were zero with a mean of 11.14, maximum of 2264, and a standard deviation of 51.8.
(TIFF)

S2 Fig Nominal 95% prediction intervals of larvae numbers. Here we show the calculated 95% prediction intervals for each observation in the (a) training set and the (b) test set using the bootstrap method (Section). These are plotted in the increasing order of the upper bound of the prediction intervals for clarity. The nominal 95% prediction intervals of larvae numbers produced a coverage probability of 98.18% and 96.36% for the training and for test sets respectively.
(TIF)

S1 Table Comparison of various regression models for stage 2. We used a zero-inflated negative binomial regression model to estimate the number of larvae in stage 2 of our approach. While the over-dispersed larvae counts with a large number of zeroes suggests the suitability of this regression model we also fitted several other models which were then statistically compared. We used the same set of candidate predictor terms and used a two-way stepwise selection method to choose which terms should be included in the model. First, using a likelihood ratio test, we revealed Poisson regression outperforms a multiple linear regression (p-value <2.2e-16). Vuong’s closeness test was used to compare the Poisson regression model, negative binomial regression model, zero-inflated Poisson regression model and zero-inflated negative binomial regression model. The Bayesian Information Criterion (BIC) -corrected Vuong statistic and the corresponding p-values are given in this table. It can be seen that zero-inflated negative binomial model outperforms the others.
(PDF)
