# Supplementary figures and images for: Risk prediction system for dengue transmission based on high resolution weather data
Source: PLoS One. 2018 Dec 6;13(12):e0208203. doi: 10.1371/journal.pone.0208203 (PMC6283552; doi:10.1371/journal.pone.0208203)

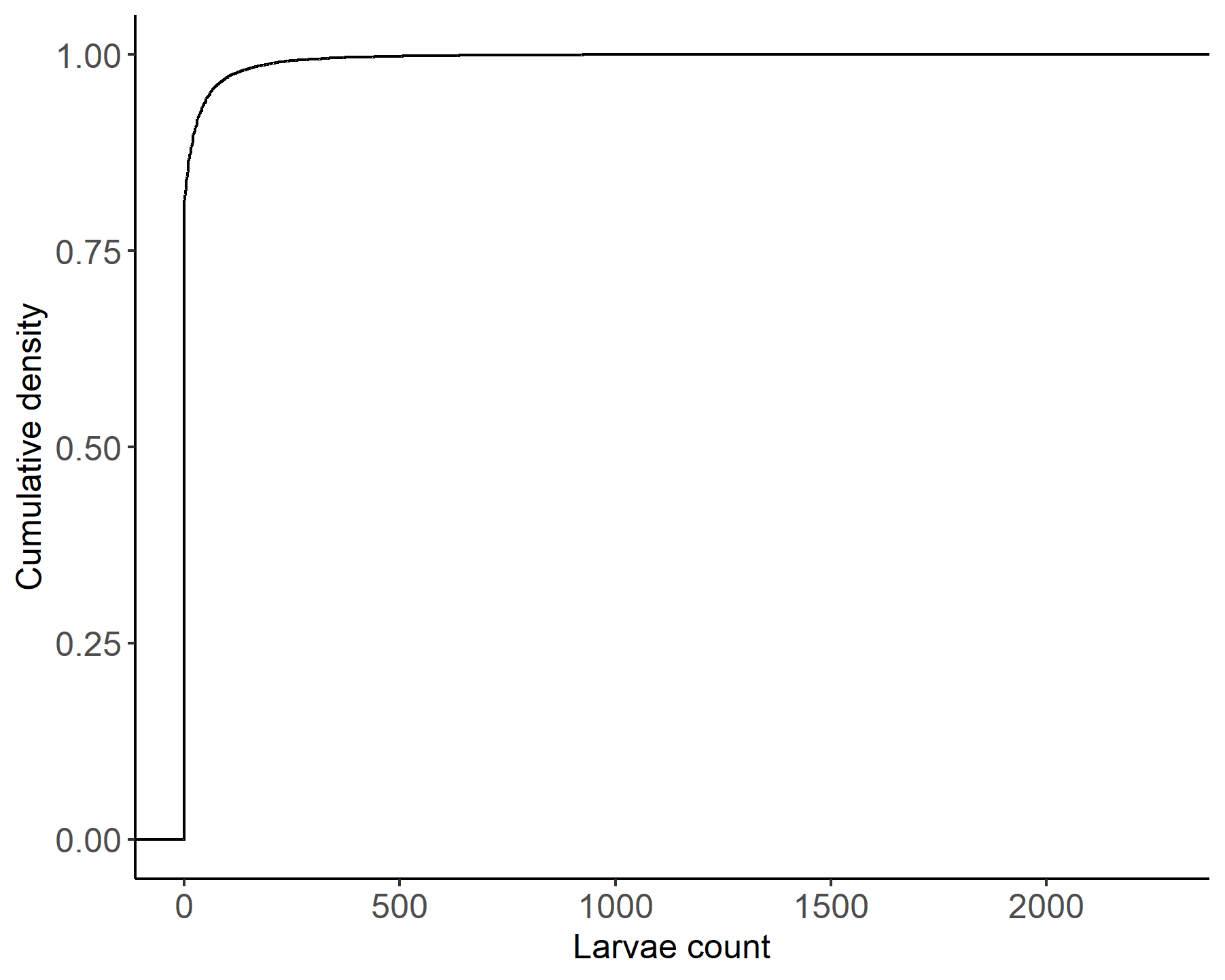

Supplement: S1 Fig — The observed numbers of Aedes aegypti larvae consist of a large number of zero observations and it follows a positively skewed distribution with a high variance. The minimum and median of the counts were zero with a mean of 11.14, maximum of 2264, and a standard deviation of 51.8. (TIFF) [file pone.0208203.s001.tiff]

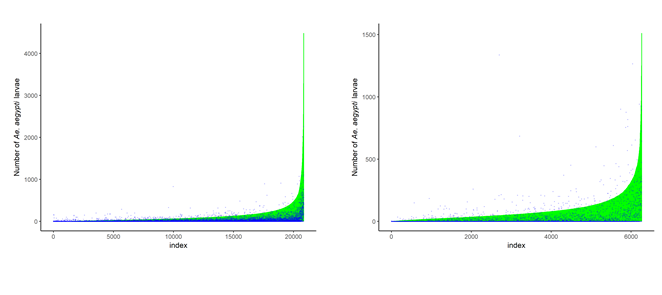

Supplement: S2 Fig — Here we show the calculated 95% prediction intervals for each observation in the (a) training set and the (b) test set using the bootstrap method (Section). These are plotted in the increasing order of the upper bound of the prediction intervals for clarity. The nominal 95% prediction intervals of larvae numbers produced a coverage probability of 98.18% and 96.36% for the training and for test sets respectively. (TIF) [file pone.0208203.s002.tif]
