# Supplementary material for: Risk prediction system for dengue transmission based on high resolution weather data
Source: PLoS One. 2018 Dec 6;13(12):e0208203. doi: 10.1371/journal.pone.0208203 (PMC6283552; doi:10.1371/journal.pone.0208203)
Supplement: S1 Table — We used a zero-inflated negative binomial regression model to estimate the number of larvae in stage 2 of our approach. While the over-dispersed larvae counts with a large number of zeroes suggests the suitability of this regression model we also fitted several other models which were then statistically compared. We used the same set of candidate predictor terms and used a two-way stepwise selection method to choose which terms should be included in the model. First, using a likelihood ratio test, we revealed Poisson regression outperforms a multiple linear regression (p-value <2.2e-16). Vuong’s closeness test was used to compare the Poisson regression model, negative binomial regression model, zero-inflated Poisson regression model and zero-inflated negative binomial regression model. The Bayesian Information Criterion (BIC) -corrected Vuong statistic and the corresponding p-values are given in this table. It can be seen that zero-inflated negative binomial model outperforms the others. (PDF) [file pone.0208203.s003.pdf]

Comparison of various regression models for stage 2.

Model 1	Model 2	Vuong	p-value
Negative binomial	Poisson	57.27	<2.2e-16
Zero-inflated Poisson	Poisson	56.55	<2.2e-16
Zero-inflated negative binomial	Negative binomial	5.91	1.75e-09
Zero-inflated negative binomial	Zero-inflated Poisson	30.92	<2.2e-16
